# Misconceptions about traumatic brain injury among nursing students in India: implications for nursing care and curriculum

**DOI:** 10.1186/s12912-019-0388-1

**Published:** 2019-12-09

**Authors:** Jothimani Gurusamy, Sailaxmi Gandhi, Senthil Amudhan, Kathyayani B. Veerabhadraiah, Padmavathi Narayanasamy, Sunitha T. Sreenivasan, Marimuthu Palaniappan

**Affiliations:** 1 College of Nursing, National Institute of Mental Health And Neuro Sciences (NIMHANS), Bangalore, 560029 India; 2 Department of Nursing, National Institute of Mental Health And Neuro Sciences (NIMHANS), Bangalore, 560029 India; 3 Department of Epidemiology, NIMHANS, Bangalore, India; 4 Department of Biostatistics, NIMHANS, Bangalore, India

**Keywords:** Misconceptions, Traumatic Brain Injury, Prevention, Educational Intervention, Nursing Education, Nursing Care

## Abstract

**Background:**

Despite the devastating consequences of Traumatic brain injuries (TBIs), TBI misconceptions are common among healthcare professionals. As an essential member of multi-professional team providing TBI care, it is important that nurses have correct information and adequate skills to achieve the best possible outcomes for TBI. For example, some common misconceptions about TBIs are that a second blow to the head can improve memory functioning and wearing seatbelts can cause as many brain injuries as it prevents. In India, perhaps such misconceptions towards TBI among nursing professionals were not yet documented. As nursing students form the future health workforce, understanding TBI misconceptions among nursing students in resource-limited settings like India will provide useful information for strengthening the nursing curricula for improved care and rehabilitation of TBIs.

**Methods:**

We used a cross-sectional survey to study the TBI misconceptions among nursing students in India. A Common Misconceptions about Traumatic Brain Injury (CM-TBI) questionnaire was administered to 154 nursing students from a nursing college of a tertiary care neuro-centre in India. The mean percentage of misconceptions were calculated for 7-domains of CM-TBI. T-test for independent samples and ANOVA were used to study the association of misconception with socio-demographic variables using total score for each respondent.

**Results:**

Of the 143 nursing students who completed the survey, majority of them were female (97%) and in the 19-20 year age-group (95.1%). Domain on brain damage (81.1%) had highest rate, while amnesia domain (42.0%) had lowest rate of misconception. The overall mean-score was 22.73 (Standard Deviation**:** 4.69) which was significantly higher than the median score of 19.5. The study did not show significant differences on overall misconceptions about TBI for any of the socio-demographic characteristics.

**Conclusions:**

Misconceptions about TBIs were common among nursing students and it was pervasive irrespective of age, gender, place of residence and year of education. A need to strengthen nursing curriculum in the area of TBIs has been emphasized for improved care and management of TBIs. The study findings also suggest the need for understanding such misconceptions among other healthcare professionals involved in TBI care.

## Background

Traumatic brain injuries (TBIs) are a major public health challenge globally. Globally, TBIs cause non-fatal health loss across various levels of income, locations, and the lifespan, and contribute to substantial proportion of global injury burden [1]. According to World Health Organization, TBI will be one of the major cause of death and disability by the year 2020 [2]. TBI usually manifests as a complex injury leading to higher rates of residual disabilities after one-year of hospitalization [3]. In India, TBIs were leading cause of morbidity, mortality, disability and socio-economic losses. TBI was found to account for 24% of all injuries in a hospital based study from Bangalore, India [4]. A silent epidemic of TBIs has taken hold in India, as it progresses to greater growth and development in terms of motorization and urbanization [5].

Despite the burden and consequences of TBIs, a lack of knowledge and misconceptions about TBIs were common among people with TBIs and their family members [6]. Misconceptions are ideas or opinions which cause the individual to incorrectly understand ideas, objects or events, and can be generally described as a mistaken belief or a myth about specific concept [7]. For example, a common misconception about TBIs usually relate to memory functioning and seatbelt use. It is perceived that a second blow to the head can improve memory functioning and wearing seatbelts causes as many brain injuries as it prevents [8–12]. Other misconceptions include the view that people with brain injury cannot remember who they are and not recognise others and a belief that complete recovery is possible for severe brain injury [13–15]. These misconceptions can have numerous negative effects on people with TBI and their course of rehabilitation. Studies showed that people with TBI and their families had such misconceptions and misunderstanding leading to feelings of inadequacy and frustration, thereby impacting the treatment outcomes [8, 9].

Surprisingly, misconceptions about TBI were documented across various settings among healthcare providers involved in the care of patients with TBI [10, 16–18]. Besides affecting the quality of care, they pass their misconceptions to their patients, family members and general public who are dependent on them for accurate information. Considering community reintegration as one goal of rehabilitation, such misunderstandings may also slow, or even set back the progress of the person’s recovery.

Among the various health professionals (nurses, physicians, psychiatrists, psychologists, and social workers) involved in the effective care and management of TBIs, nursing professionals play a crucial role in the assessment, and management of patient with TBIs [19]. They help patient and caregivers for the reintegration into their community through effective communication and consultations with various community agencies and other health care providers. However, when they have inaccurate beliefs about TBI, they communicate inaccurate information to the patients and their families [17]. In our literature review for misconceptions about TBI patients among nurses and nursing students, several inaccurate beliefs about recovery, unconsciousness, and amnesia related to TBI were found [17, 20, 21]. This would result in poor patient and family education that will have adverse implications not only on the quality of care and recovery but also on the prevention and rehabilitation aspect of TBI.

In India, perhaps such TBI misconceptions among nursing professionals were not yet documented. With over 1 million survivors in India requiring TBI care, poor awareness and misconceptions among health care professionals had placed a great strain on the country’s already overstretched health-care system [5]. Nursing students often form the first point of contact in the healthcare system and the future health workforce in India. Educating them through the regular curricula will be a more effective and sustainable strategy to tackle the worsening TBI crisis in India when compared to educating nurses through special training and continuing education. Hence, understanding such misconceptions about TBI among nursing students in resource-limited settings like India will provide valuable evidence to design and strengthen the nursing curricula for improved care and rehabilitation of TBI.

### Aim

The objectives of this study are (1) to assess the misconceptions about traumatic brain injury among nursing students (2) to study the association of overall misconception about traumatic brain injury with socio-demographic characteristics of nursing students.

## Methods

We used a cross-sectional study design in a nursing college attached to tertiary care neuro-centre in India. A convenience sample of 154 nursing students was used for the present study. The study participants were recruited between July to August 2016. Undergraduate nursing students in 2^nd^ and 3^rd^ year who had completed the theoretical instructions in trauma care nursing and who also had attended clinical practicum in surgical and emergency care unit were included for the present study. This was to ensure that nursing students had enough exposure to TBI care.

### Sample size

With an intake of 77 nursing students per year, the size of the population of nursing students in 2^nd^ and 3^rd^ year in the nursing college was 154. We hypothesized a prevalence of 50% (that gives a maximum sample size) for TBI misconceptions among nursing students. With 95% confidence level and a margin of error that did not exceed ±5%, a sample size of 111 would be required for a population of 154. The sample size was estimated using the equation [22]

$$ \boldsymbol{n}=\left[\mathbf{DEFF}\ast \mathbf{Np}\ \left(\mathbf{1}-\mathbf{p}\right)\right]/\left[\right({\mathbf{d}}^{\mathbf{2}}/{{\mathbf{Z}}^{\mathbf{2}}}_{\mathbf{1}-\boldsymbol{\upalpha} /\mathbf{2}}\ast \left(\mathbf{N}-\mathbf{1}\right)+\mathbf{p}\ast \left(\mathbf{1}-\mathbf{p}\right)\Big]\mathrm{N}=\mathrm{Population}\ \mathrm{Size}\ \left(\mathrm{for}\ \mathrm{finite}\ \mathrm{population}\ \mathrm{correction}\ \mathrm{factor}\ \mathrm{or}\ \mathrm{fpc}\right)\mathrm{p}=\mathrm{Hypothesized}\%\mathrm{frequency}\ \mathrm{of}\ \mathrm{outcome}\ \mathrm{factor}\ \mathrm{in}\ \mathrm{the}\ \mathrm{population}\mathrm{d}=\mathrm{confidence}\ \mathrm{limit}\ \mathrm{as}\%\mathrm{DEFF}\ \left(\mathrm{Design}\ \mathrm{Effect}\right)=1 $$

Considering the non-response, it was decided to include all the 154 students for the present study.

### Data collection

After getting permission from the Principal of College of Nursing, a written informed consent was obtained from the students. Participation was voluntary and no monetary stipend, incentive, or individual feedback was offered to any participant. After giving a brief explanation about the study, the questionnaire was administered to 154 nursing students at the end of their regular classes. Anonymity was maintained, as no names or protected health information was entered into the survey. Participants were asked to read and anonymously complete a 40-item, self-report questionnaire that was designed to assess the knowledge about head trauma, its effects, recovery, and so forth. Participants were to choose between the answers ‘true’, ‘probably true’, ‘probably false’, and ‘false’ for each item. The entire process took less than 40 minutes for each participant. Of the 154 students surveyed, 11 students with incomplete questionnaire were excluded and a final sample of 143 students with complete response were included, yielding a response rate of 92.9%.

### Study Instrument

The study instrument used for the present study had two parts. The first part consisted of a socio demographic schedule with 8 items, and the second part consisted of Common Misconceptions about Traumatic Brain Injury (CM-TBI) questionnaire. CM-TBI is a 40-item self-report questionnaire with seven key domains namely Prevention, Brain damage, Brain injury sequelae, Unconsciousness, Amnesia, Recovery process and Rehabilitation. For each item under CM-TBI, the participants had to respond on a 4-point Likert Scale (‘true’, ‘probably true’, ‘probably false’, and ‘false’) to indicate their agreement or disagreement. Out of the 40 items, 24 items were developed by Gouvier *et al*. and the remaining 16 items were based on the clinical expertise of Pappadis [8, 11].

Variations of CM-TBI questionnaire has been used in several studies to assess TBI misconceptions in the general population, educational professionals, rehabilitation staff, and nursing students [9, 10, 12–15, 17, 23]**.** We used the present 40-item CM-TBI questionnaire for the following reasons (1) the 40-item CM-TBI questionnaire allows for comprehensive assessment when compared to other shorter variations [13–15, 17]. (2) the 40-item CM-TBI was reported to have good reliability and internal consistency with a Cronbach’s α ranging from 0.69 to 0.84 [8, 24]. (3) A short variant of present 40-item CM-TBI questionnaire was used already for nursing students [17]. (4) Swift and Wilson (2001) found that medical professionals who do not specialize in brain injury had many misconceptions that were similar to general public [6]. The utility of CM-TBI questionnaire among both general public and nursing students would allow for such valid comparisons (between general public and nursing students). (5) More importantly, any questionnaire developed specifically for nursing students would have led to medicalising the problem without much relevance for public health education. The 40-item CM-TBI questionnaire has merit and scope in providing valuable information on the health education needs of nursing students, who also assumes a critical role of health communicator when providing care for TBIs.

Prior to administration, the questionnaire was validated independently by three experts in the field of nursing education and public health for clarity, relevance and content. Two scoring schemes were proposed for CM-TBI, namely a dichotomized categories of “true” or “false” (probably true is considered true; probably false is considered false) and a stringent 4-point scale (any response other than absolutely true or false is considered incorrect) [25]. Numerous published studies have used dichotomized categories for scoring [13, 17, 23, 24]. A Cronbach’s α of 0.95 was reported for dichotomised categories while the Cronbach’s α was 0.33 when the stringent 4-point scale was used. Therefore, dichotomised scoring was used for the current study.

### Data analysis

Data from the questionnaire responses were coded and entered into the database and analysed using the Statistical Package for Social Sciences, Version 18.0 (SPSS Inc., Chicago, Ill., USA). All demographic data were analyzed using frequencies and percentages that described the sample. The response to 4-point likert scale was dichotomised into “true” or “false” with true representing ‘true’ and ‘probably true’ response and false representing ‘false’ and ‘probably false’ response. The dichotomised response was then scored as “1” for misconception and “0” for correct response. Total score was calculated for each respondent with higher score indicating higher misconception. The percentage of participants with misconception for each item and the mean percentage of participants with misconception for each questionnaire domain was calculated.

Before analyzing our second objective to study the association of socio-demographic characteristics with misconception, we computed Cronbach’s α to determine the internal consistency of entire questionnaire in our study sample. The t-test for independent samples and ANOVA (Analysis of Variance) were used to study the association of misconception and socio-demographic variables using total score for each respondent.

With no cut-off defined for CM-TBI, we arbitrarily hypothesized a conservative cut-off that corresponded with the median value of total CM-TBI score to suggest the need for intervention. The mean score on CM-TBI for the sample was then compared with our arbitrary cut-off using one-sample t-test. P-value <0.05 was considered as statistically significant. To overcome the non-normality of our sample distribution and to increase the robustness of our estimates, we used bootstrapping for our data analysis using1000 random bootstrap samples with an alpha of 0.05 [26].

### Results

#### Sample characteristics

Of the 143 participants who completed the survey, majority were female (96.5%) and belonged to 19-20 age-group (95.1%). Eighty-three percent of them were Christians and belonged to nuclear family. Rural residents (51.7%) were slightly more than urban residents and students of II-year (53.8%) were slightly more than III-year students. Nearly 33% had witnessed their friends and others who sustained brain injury (Table 1).

**Table 1:** Demographic characteristics of the participants *N*=143

Demographic variables	n (%)
Age (years)	
17-18	7 (4.9)
19-20	136 (95.1)
Gender	
Female	138 (96.5)
Male	5 (3.5)
Religion	
Christian	119 (83.2)
Hindu & others	24 (16.8)
Type of family	
Nuclear	119 (83.2)
Joint	24 (16.8)
Place of residence	
Urban	32 (22.4)
Rural	74 (51.7)
Semi-urban	37 (25.9)
Vehicles owned	
Bicycle	35 (24.4)
2 &4-wheeler	82 (57.4)
No	26 (18.2)
Prior exposure to Brain injury	
Yes	96 (67.1)
No	5
47 (32.9)	6

#### Internal Consistency

The Cronbach’s α for the 40-questionnaire items in our study was α = 0.680. Two-items namely “People who have had one brain injury are more likely to have a second one” and “A person who has a brain injury will be ‘just like new’ in several months” were ambiguous and misleading due to cultural differences in semantics. Eliminating these two items from the recovery domain yielded an alpha of 0.698 (0.622 - 0.765) indicating acceptable internal consistency. For further analysis, 38 questionnaire items were used.

#### Misconceptions about TBI

Misconceptions varied across the 7 domains. Domain related to brain damage (mean percentage: 81.1) had highest rate of misconception followed by brain injury sequelae (mean percentage: 74.7) (Table 2). While prevention domain (mean percentage: 62.2) had modest rate of misconception, the lowest rate of misconception was observed for amnesia domain (mean percentage: 42).

**Table 2:** Percent misconceptions among nursing students for traumatic brain injury

Category	Items	Item-wise correct response	Respondents With misconceptions n (%)
Prevention	1.You don’t need seatbelts as long as you can brace yourself before a crash	F	125 (87.4)
	2.It is more important to use seatbelts on long trips than in driving around town	F	53 (37.1)
	3. It is safer to be trapped inside a wreck than to be thrown clear	T	94 (65.7)
	4. Wearing seatbelts causes as many injuries as it prevents	F	84 (58.7)
	**Mean % of misconceptions**		**62.2**
Brain damage	5. A head injury can cause brain damage even if the person is not knocked out	T	122 (85.3)
	6. A little brain damage doesn’t matter much, since people only use a part of their brains anyway	F	126 (88.1)
	7. It is obvious that someone has brain damage because they look different from people who don’t have brain damage	F	97 (67.8)
	8. Whiplash injuries to the neck can cause brain damage even if there is no direct blow to the head	T	118 (82.5)
	**Mean % of misconceptions**		**80.9**
Brain injury sequelae	9. It is common for people with brain injuries to be easily angered	T	81 (56.6)
	10. It is possible that a person’s personality will change after a brain injury	T	104 (72.7)
	11.Problems with speech, coordination, and walking can be caused by brain damage	T	131 (91.6)
	12. Problems with irritability and difficulties controlling anger are common in people who have had a brain injury	T	117 (81.8)
	13. Most people with brain damage are not fully aware of its effect on their behaviour	T	123 (86.0)
	14. Brain injury patients usually show a good understanding of their problems because they experience them every day	F	62 (43.4)
	15. Brain injuries may cause one to feel depressed, sad and hopeless	T	118 (82.5)
	16. Drinking alcohol may affect a person differently after a brain injury	T	116 (81.1)
	17. It is common for people to experience changes in behaviour after a brain injury	T	110 (76.9)
	**Mean % of misconceptions**		**74.7**
Unconsciousness	18. When people are knocked unconscious, most wake up quickly with no lasting effects	F	66 (46.2)
	19. People in a coma are usually not aware of what is happening around them	T	116 (81.1)
	20. Even after several weeks in coma, when people wake up, most recognise and speak to others right away	F	48 (33.6)
	**Mean % of misconceptions**		**53.6**
Amnesia	21. People usually have more trouble remembering things that happen after an injury than remembering things from before	T	115 (80.4)
	22. Sometimes a second blow to the head can help a person remember things that were forgotten	F	61 (42.7)
	23. A person with a brain injury may have trouble remembering events that happened before the injury, but usually does not have trouble learning new things	F	35 (24.5)
	24. People with brain injury can forget who they are and not recognise others, but be normal in every other way	F	29 (20.3)
	**Mean % of misconceptions**		**42.0**
Recovery	25.Recovery from a brain injury usually is complete in about 5 months	F	80 (55.9)
	26. Complete recovery from a severe brain injury is not possible, no matter how badly the person wants to recover	T	81 (56.6)
	27. Once a person is able to walk again, his/her brain is almost fully recovered	F	63 (44.1)
	28. Slow recovery may continue even 1 year after injury	T	119 (83.2)
	29. It is necessary for a person to go through a lot of physical pain to recover from a brain injury	F	30 (21.0)
	30. Once a person with a brain injury realises where they are, they will always be aware of this	F	44 (30.8)
	31. A person who has recovered from a head injury is less able to withstand a second blow to the head	T	106 (74.1)
	32. Asking persons who have had a brain injury about their progress is the most accurate, informative way to find out how they have progressed	F	38 (26.6)
	33. It is good advice to remain completely inactive during recovery from a brain injury	F	83 (58.0)
	34. Once a person recovering from a brain injury feels ‘back to normal’ the recovery process is complete	F	38 (26.6)
	35. How quickly a person recovers depends mainly on how hard he or she works at recovering	F	29 (20.3)
	**Mean % of misconceptions**		**45.2**
Rehabilitation	36. ‘Cognitive’ refers to thinking processes such as memory, attention and learning	T	135 (94.4)
	37. ‘Cognitive’ refers to the ability to move your body	F	106 (74.1)
	38. The primary goal of brain injury rehabilitation is to increase physical abilities such as walking	F	48 (33.6)
	**Mean % of misconceptions**		**67.4**

The entries in boldface represent the mean%

Item-wise analysis showed that item “Problems with speech, coordination, and walking can be caused by brain damage” under brain sequelae domain had highest rate of misconception (91.6%), while the item “People with brain injury can forget who they are and not recognise others, but be normal in every other way” under amnesia domain and the item “How quickly a person recovers depends mainly on how hard he or she works at recovering” under recovery domain had lowest rate of misconception (20.3% each) (Table 2).

#### Association of misconceptions with socio-demographic characteristics

The overall mean score for our sample was 22.73 with Standard Deviation (SD) of 4.69 and this was significantly higher than the hypothesized population mean of 19.5 (t_(142)_=8.249, p-value=0.001) (Table 3). The study did not show significant differences for the overall misconceptions about TBI, when the participants were classified by age, gender, religion, year of education, type of family, place of residence, vehicles owned and prior exposure to brain injury (Table 3).

**Table 3:** Association of socio-demographic variables with CM-TBI

Demographic variables	n	CM-TBI score		
		Mean ± SD	t/F	P-value
Age (years)				
17-18	7	21.29 ± 5.99	0.837	0.404
19-20	136	22.81 ± 4.63		
Gender				
Female	138	22.76 ± 4.64	0.355	0.784
Male	5	22.00 ± 6.52		
Religion				
Christian	119	22.76 ± 4.61	0.125	0.899
Hindu & others	24	22.63 ± 5.19		
Year of Education				
Second	77	22.17 ± 4.69	1.566	0.117
Third	66	23.39 ± 4.64		
Type of family				
Nuclear	119	22.75 ± 4.70	0.077	0.939
Joint	24	22.67 ± 4.72		
Place of residence				
Urban	32	21.88 ± 4.98	1.075	0.344
Rural	74	23.26 ± 4.79		
Semi-urban	37	22.43 ± 4.19		
Vehicles owned				
Bicycle	35	22.26 ± 4.43	2.620	0.076
2 &4-wheeler	82	22.34 ± 4.74		
No	26	24.62 ± 4.59		
Prior exposure to brain injury				
Yes	47	22.85 ± 4.74	0.208	0.827
No	96	22.68 ± 4.69		
Overall Sample	143	22.73 ± 4.69	8.249*	0.001*

*p <0 .05, based on one sample t-test on 1000 bootstrap samples using 19.5 (median for total score of 38) as cut-off for the population mean

## Discussion

Knowledge about TBIs and its effects among health care professionals are very crucial to provide proper care to the patients and their family. Misconceptions related to TBI are widespread and are even demonstrated in the medical field [6]. Reaffirming the same, the present study had found considerable misconceptions about TBIs among nursing students. To the best of our knowledge, the current study is the first to document nursing student’s misconceptions regarding TBI. With nursing curriculum requiring 50% as passing grade in India, misconceptions can be arbitrarily defined acceptable for a score upto 19.5 (median score for 38 items). In our study, the overall mean misconception score was significantly higher than the median score of 19.5 and nearly 77.6% of our sample had alarming scores (>19.5) for misconception. Even with the liberal cut-off, the nursing students had a significantly higher misconception when compared to the maximum allowable misconception, indicating the enormity of TBI-misconceptions and the need for immediate intervention among nursing students. The domains that had highest rate of misconceptions were brain damage (81%) and brain injury sequelae (74.7%). This was quite contrast to the findings from lay public (8.3%), correctional healthcare professionals (6.1%) and from nursing students (6.6%) which reported fewest misconceptions in brain damage category [17, 27, 28]. Even though the rates of misconceptions were relatively low for amnesia (42.0%) and recovery (45.2%) domains in our study, they were comparable or even higher when compared to earlier studies from general public and other health care professionals from other countries [13–15, 17, 27, 28].

Similarly, in contrast to findings from earlier studies, the participants in the present study had relatively high rates of misconception regarding seatbelt (87.4% -37.1%) indicating a poor knowledge of seatbelt use related to motor vehicle accidents [17]. In particular, about 87% of the participants incorrectly agreed with the statement: “You don’t need seatbelts as long as you can brace yourself before a crash”. Surprisingly similar to our present study, a variety of educational and health care professionals also had some of the same misconceptions as the general public in areas such as length and extent of recovery, diversity of sequelae, and in specific domains such as seatbelt use [6, 10, 17–19, 29]. This highlights the need to focus on dispelling the incorrect ideas about seatbelt (which are proven to be simple and effective prevention of TBIs) among nursing students so that they can pass correct information to the family of patients with TBI and subsequently to the general public.

### Implications for Nursing Care

Overall, the type and degree of misconceptions held by nursing students in the present study were quite different (with higher rates) when compared to narratives from other developed countries and has the potential to adversely impact not only clinical care but also on recovery and rehabilitation. Responses from TBI patients and caregivers indicated that health professionals who did not specialize in brain injury had same misconceptions as lay people regarding the extent of TBI recovery and deficits. They even misidentified persons with TBI as learning disabled or mentally ill and subsequently failed to provide necessary support for their proper recovery and rehabilitation [6]. In resource-limited settings like India, nursing students are future nurses providing primary care in many settings. They often have initial contact with survivors and hence they should be able to accurately recognize and/or diagnose TBIs with adequate knowledge about when and where to refer these individuals for further treatment [30].

Consistent with other studies, we did not find personal experience with TBI (self, family member) to influence misconceptions [11, 17]. Unlike other studies, we did not find significant differences for other socio-demographic variables like age, gender, place of residence, etc., for misconceptions. This highlights the pervasiveness of this problem and also suggests the need for understanding such misconceptions among other healthcare professionals involved in TBI care.

Since the participant nursing students have significant practicum exposure, the possibility of receiving wrong information through practices by nurses in the ward cannot be ruled out [17]. This high level of misconceptions in the present study indicates the need to improve the theoretical and practical skills among nurses who provide TBI care from whom the nursing students learn. Specifically, nurses providing TBI care and nursing teachers/instructors may need to seek educational experience to update the recent advances in patient care related to TBIs through continuing nursing education, attending workshops, and conferences for their clinical competence so that they can provide appropriate TBI care as well as practical support and mentoring to the trainee-nurses.

### Implications for Nursing Curriculum

In India, Indian Nursing Council is the statutory body that regulates and guide all nursing curriculum syllabi to ensure that the Registered Nurses have the required core competencies. On review, the nursing curriculum for neurological disorders include materials on anatomy, physiology, common conditions of neurological disorders, nursing assessment, medical and surgical nursing management, patient communication, special therapies, therapeutic agents and the implications of nursing through various teaching and learning activities [31]. The curriculum mainly focuses on typical education and training to prepare junior nurses to provide effective care in general practice settings.

The results of the study indicated that nursing students had considerable misconceptions towards TBIs. In future, nursing students are going to play a vital role in the delivery of health care services for patients in assessing and treating TBI-related sequelae. Early career nursing professionals such as undergraduate nursing students often have eagerness to learn information. This makes their training period an ideal time to provide suitable educational measures for TBIs. Based on the results of this study that suggest the pervasiveness of misconceptions about TBI among nursing students, a need to strengthen the nursing curricula and syllabi on TBI has been emphasized. Such curricula should focus on improving clinical and theoretical instructions by adding a specific module/model concept on TBI so that nursing professionals have adequate educational experience to acquire correct knowledge and skills in this area. To pass correct information to the victim family, the instruction modules should have adequate emphasis on communication and counselling skills.

The existing curriculum in particular, does not adequately acknowledge the nurse’s beliefs. Nurse’s beliefs were found to have strong influence on nursing care and health teaching for patients and families [32]. The nursing curricula therefore must be customized to the local educational needs of the nursing students and focus on cultural and personal beliefs to help dispel the myths held by students and to clarify the reasons for such TBI misconceptions among patients or nurses. The common TBI misconceptions identified in the present study could be utilized for designing the contents of TBI nursing and for the development of specific modules/educational intervention programmes on TBI for nursing students in India.

Besides the regular training, they can be targeted at critical time points of their professional’s career; for example, educational resources may be provided to early career nursing professionals as well as to primary care providers who are likely to have first contact with survivors. For those in their late-careers, specific resources like universities and colleges that provide education and training for brain injury professional in various disciplines may be used.

### Global relevance for nursing curriculum and care

Recent evidences across the globe had documented misconceptions related to TBI in various settings (including nursing students) and emphasized the effectiveness of educational interventions in addressing TBI-related misconceptions [13–16, 20, 23, 33–37]. The numerous and substantial misconceptions found in the current sample of nursing students were comparable or even higher than the recent evidences from various settings. With significant consequences of TBI misconceptions towards recovery and prevention, documentation of such TBI misconceptions across various settings indicate the importance of TBI misconceptions towards global reduction of TBI burden [30].

Specifically, the findings of the study have implications for nursing practice and education globally. With an increase in global incidence and prevalence of TBI, the number of patients with TBI who seek care from nurses on a regular basis are going to increase and it is important that nurses have adequate knowledge and information to provide care and education to patient, family and community [1]. Owing to huge population, addressing TBI misconceptions among nursing students in India will be crucial to make global differences in the burden of TBI. The increasing investment in theoretical knowledge may prove to be ineffective, if common misconceptions are not targeted in student learning [38]. A need to acknowledge nurse’s beliefs has been emphasized for nursing practice and education [32]. The existing nursing curricula should focus on increasing reflective and critical thinking of the beliefs in the theory and practical system to rectify their misconceptions to pursue excellence in the working world [32]. Future researches should focus on developing educational strategies relevant for TBI misconceptions among nursing students and examine their effectiveness.

### Limitations

We acknowledge the limitations of our study. The present study was cross-sectional with known limitations. However, the study is adequate for establishing the magnitude of the problem. Bootstrapping was use to overcome the non-normality of our sample distribution. The nursing students in the present study are sampled conveniently from a nursing college attached to tertiary care neuro-centre in India. As these students are expected to have higher exposure to TBIs, it is highly likely that the misconceptions are expected to be higher in other educational settings for nursing students with limited exposure. The study used dichotomized scoring scheme which might have under-estimated the misconceptions when compared to more stringent scoring scheme where uncertain correct answers were scored as incorrect. The study instrument was distributed by the researcher who is a senior post-graduate scholar, not directly involved in teaching nursing students. As the study instrument was self-administered, there is no possibility for interviewer bias. However, the influence of social desirability bias in self-reporting cannot be ruled out completely even for assessing TBI misconceptions. Notwithstanding these limitations, the present study is the first of its kind in India to throw light on the misconceptions about TBIs among nursing students which are critical for strengthening the nursing curriculum for TBI care.

## Conclusion

The present study is perhaps the first attempt in India to understand the nature of TBI misconceptions among nursing students and highlighted the pervasiveness of such misconceptions in Indian context. In future, they are the professionals who will work closely with individuals and their families with TBI. Despite the high burden of TBIs in India, nursing professional trainees are not usually informed about the physical, behavioural, cognitive and psychosocial consequences of TBIs. This will have potential impact on the prevention, care, recovery and rehabilitation of TBIs. Thus, the study findings emphasize the need to provide educational experience to nursing students through strengthening of nursing curriculum and syllabi in the area of TBIs. This will be one of the important steps towards improving the quality of services related to TBIs.

## Data Availability

The datasets used and/or analysed during the current study are available from the corresponding author upon a reasonable request.

## Abbreviations

ANOVA: Analysis of Variance
CM-TBI: Common Misconceptions about Traumatic Brain Injury
DEFF: Design Effect
NIMHANS: National Institute of Mental Health And Neuro Sciences
SD: Standard Deviation
TBI: Traumatic Brain Injury
